# *In vitro* and *in vivo* enhancement of colistin activity against *Escherichia coli* strains by macelignan

**DOI:** 10.1128/spectrum.01129-26

**Published:** 2026-06-10

**Authors:** Lian Liu, Tianle Pan, Ruyue Huang, Xueling Liao, Mi Wang, Chengzhong Fei, Liwei Guo, Yingchun Liu

**Affiliations:** 1 China College of Animal Science and Technology, Yangtze University 47897, Jingzhou, Hubei, China; 2 Shanghai Veterinary Research Institute, Chinese Academy of Agricultural Sciences 118161, Shanghai, China; 3 Shanghai Gongyi Pharmaceutical Co., Ltd. 267439 , Shanghai, China; JMI Laboratories, North Liberty, Iowa, USA

**Keywords:** macelignan, natural product, colistin, *Escherichia coli*, synergistic antibacterial, antibacterial mechanism

## Abstract

Colistin remains a last-line antibiotic against multidrug-resistant Gram-negative pathogens, but its clinical utility is limited by nephrotoxicity and emerging resistance. This study identifies macelignan, a natural lignan from nutmeg, as a potent synergist that enhances colistin activity against *Escherichia coli* (*E. coli*) through a novel metabolic disruption mechanism. *In vitro*, macelignan reduced the colistin minimum inhibitory concentration (MIC) by approximately 100-fold, with the combination (8 µg/mL macelignan + 0.008 µg/mL colistin) achieving complete bacterial eradication within 4 h. *In vivo*, combination therapy significantly reduced bacterial burden in a mouse thigh infection model (*P* < 0.05). Mechanistic studies revealed that macelignan disrupts bacterial energy metabolism by inhibiting the citric acid cycle, leading to ATP depletion. This energy compromise impairs the bacterial capacity to repair colistin-induced membrane damage, exacerbating membrane disruption and accelerating bacterial death. These findings establish macelignan as a promising adjuvant for colistin-based therapy and elucidate a dual “internal and external pincer” mechanism that could inform future antibiotic combination strategies.

**IMPORTANCE:**

The continued efficacy of colistin, a last-line antibiotic against multidrug-resistant Gram-negative pathogens, is increasingly threatened by dose-limiting nephrotoxicity and the global spread of resistance determinants. This study introduces macelignan—a naturally occurring lignan from nutmeg—as a highly effective colistin adjuvant that operates through a previously unrecognized mechanism centered on metabolic disruption. By targeting the bacterial citric acid cycle, macelignan depletes intracellular ATP, thereby crippling the energy-dependent machinery required for membrane repair following colistin-induced damage. This dual “internal and external pincer” strategy not only dramatically reduces the colistin concentration needed for bactericidal activity but also significantly lowers bacterial burden *in vivo*. These findings provide a mechanistic framework for developing metabolism-targeting adjuvants to revitalize last-resort antibiotics and expand the therapeutic arsenal against recalcitrant Gram-negative infections.

## INTRODUCTION

Antibiotics have fundamentally transformed the management of bacterial infections by targeting essential bacterial processes, such as cell wall synthesis, protein production, and membrane integrity (1). Their introduction led to a dramatic decline in infection-related mortality, establishing them as a cornerstone of modern medicine. However, the widespread and often indiscriminate use of antibiotics has accelerated the emergence of bacterial resistance, now recognized as a major global public health threat (2, 3).

Colistin (COL), a narrow-spectrum polymyxin antibiotic, retains potent activity against most Gram-negative pathogens, including *Pseudomonas aeruginosa*, *Acinetobacter baumannii*, and *Escherichia coli*, positioning it as a last-line therapeutic option for multidrug-resistant (MDR) infections (4). Its bactericidal mechanism involves electrostatic interaction between its cationic peptide moiety and the anionic lipid A component of lipopolysaccharide (LPS), displacing divalent cations from the outer membrane and ultimately leading to cell lysis (5, 6). Despite its efficacy, the clinical utility of colistin is severely constrained by dose-dependent nephrotoxicity and neurotoxicity, necessitating alternative strategies to preserve its therapeutic potential while minimizing adverse effects (7).

Combination therapy has emerged as a promising and cost-effective approach to combat MDR bacterial infections, offering advantages over the lengthy and expensive process of novel antibiotic development (8, 9). Numerous studies have documented synergistic effects when colistin is combined with various antibiotics or non-antibiotic agents (10). In parallel, natural products and traditional Chinese medicine constituents have garnered increasing attention as potential sources of antibiotic adjuvants (11–13). For instance, thymol, a bioactive essential oil derived from medicinal plants, enhances colistin activity by disrupting outer membrane integrity and increasing permeability (14).

Nutmeg (*Myristica fragrans*), a tropical evergreen tree native to Indonesia and cultivated across South Asia and the Caribbean, exhibits broad pharmacological activities, including antibacterial and antifungal properties. Among its bioactive constituents, macelignan has been identified and demonstrated to possess antioxidant, anti-inflammatory, anti-caries, and hepatoprotective effects (15–17). Recent studies have further revealed that macelignan acts as a novel JAK1 inhibitor, suppressing non-small cell lung cancer growth (18).

However, despite these documented bioactivities, the potential synergistic antibacterial effect between macelignan and colistin against *E. coli* has not been investigated. In this study, we demonstrate for the first time that macelignan significantly enhances the susceptibility of *E. coli* to colistin, both *in vitro* and *in vivo*. These findings highlight a promising new therapeutic strategy for combating Gram-negative bacterial infections.

## MATERIALS AND METHODS

### Bacterial strains

The *E. coli* ATCC 25922, purchased from Shanghai Hongxin Biotechnology Co., LTD. The *E. coli* clinical isolate harboring the *mcr*-1 gene, strain M17GZZ15, is maintained at the laboratory of Shanghai Veterinary Research Institute, Chinese Academy of Agricultural Sciences. All strains were cultured in Mueller-Hinton broth (MHB) medium (Qingdao Haibo Biotechnology Company, LTD) with shaking at 37°C and stored at −80°C.

### Antimicrobial preparations susceptibility test

Colistin (CAS: 1264-72-8) and macelignan (CAS: 107534-93-0) were both commercially obtained. The Clinical and Laboratory Standards Institute guideline was used to determine the minimum inhibitory concentration (MIC) of macelignan and colistin (19). The combined antibacterial effect of macelignan and colistin was evaluated using the checkerboard method following established protocols. Briefly, single bacterial colonies were cultured in MHB and adjusted to a concentration of 10^5^ CFU/mL. Serial twofold dilutions of each antibiotic were prepared in MHB. An equal volume of bacterial suspension was then added to each well of a 96-well microtiter plate. The MIC values were defined as the lowest antibiotic concentrations with no visible bacterial growth after incubation at 37°C for 18 h. The fractional inhibitory concentration index (FICI) was calculated to quantify the interaction between the two agents. The FICI was determined as follows:

FICI = (MIC_ab_/MIC_a_) + (MIC_ba_/MIC_b_).

Where MIC_a_ and MIC_b_ represent the MICs of drug A and drug B alone, respectively; MIC_ab_ is the MIC of drug A in combination with drug B; and MIC_ba_ is the MIC of drug B in combination with drug A. An FICI value of ≤0.5 was interpreted as indicative of synergy (20).

### Time-killing assays

To further evaluate the combined antibacterial effect of macelignan and colistin *in vitro*, time-kill kinetics were determined for *E. coli* ATCC 25922 and *E. coli* M17GZZ15 carrying the mcr-1 resistance gene. The bacterial suspension with OD_600nm_ = 0.4 was placed in a sterile shaking tube and diluted 1,000 times, corresponding to approximately 1 × 10^5^ CFU/mL. Then the bacteria were treated with colistin, macelignan, and the combination of colistin and macelignan, respectively. After incubation at 37°C, 180 rpm for 0, 4, 8, 12, and 24 h, 100 μL of bacterial suspension was collected and 10-fold serially diluted in PBS, and the suspensions were plated on LBA plates. After overnight cultivation at 37°C, bacterial colonies were calculated. Three independent replicates were set for each group of experiments.

### Hemolytic assay

Add 100 μL of different concentrations of macelignan to the 96-well plate. Meanwhile, set 2% Triton-X-100 as the positive control and PBS as the negative control. Add 100 μL of 4% defibrinated rabbit blood suspension to each well and incubate at 37°C for 1 h. Finally, the supernatant was centrifuged in a 96-well plate and detected by a microplate reader.

### Membrane permeability assessment by propidium iodide staining

Membrane integrity of *E. coli* was evaluated using a propidium iodide (PI) uptake assay. Mid-logarithmic phase cultures were harvested by centrifugation (4,000 × *g*, 10 min, 4°C), washed three times with sterile PBS (pH 7.4), and resuspended to OD_600_ = 0.2. For quantitative analysis, 2 mL bacterial suspensions were transferred to 24-well plates (Corning, USA) containing 6.68 mg PI and various combined concentrations of macelignan and colistin. Fluorescence intensity was measured using a BioTek Synergy H1 microplate reader (λex = 535 nm, λem = 617 nm). For qualitative assessment, cells were co-stained with 6.68 mg PI and 4.97 mg Calcein AM (Beyotime Biotechnology, China) for 30 min at 37°C in the dark, followed by macelignan and colistin treatment for 1 h. Membrane integrity was visualized using a confocal laser scanning microscope (CLSM; Leica Microsystems CMS GmbH, Mannheim, Germany). Untreated cells stained with PI/Calcein AM served as negative controls. All experiments were performed in triplicate.

### Reactive oxygen species measurement

The levels of reactive oxygen species (ROS) in *E. coli* ATCC 25922 were measured using the fluorescence probe 2′,7′-dichlorodihydro-fluorescein diacetate (DCFH-DA) (Beyotime, China) with the excitation wavelength at 488 nm and the emission wavelength at 525 nm. In brief, the bacteria were resuspended in PBS to an OD_600_ of 0.5, then the DCFH-DA probe was added to a final concentration of 10 μM. After the bacteria were incubated at 37°C for 30 min in the dark, followed by three PBS washes to remove unbound probe. After resuscitation, cells were then treated with different combined concentrations of macelignan and colistin for 1 h under static incubation. Fluorescence intensity was measured using a BioTek Synergy H1 microplate reader (λex = 488 nm, λem = 525 nm), with ROS levels normalized to untreated controls.

### Quantitative analysis of intracellular ATP levels

Intracellular ATP content in *E. coli* ATCC 25922 was quantified using an Enhanced ATP Assay Kit (Beyotime Biotechnology, China; Cat. No. S0027) according to the manufacturer’s protocol. Mid-logarithmic phase cultures were harvested by centrifugation (4,000 × *g*, 10 min, 4°C), washed twice with sterile PBS (pH 7.4), and resuspended to OD_600_ = 0.4. Bacterial suspensions were treated with different combined concentrations of macelignan and colistin at 37°C for 1 h under static conditions. After treatment, cells were pelleted by centrifugation (12,000 × *g*, 5 min, 4°C) and lysed using lysozyme (1 mg/mL) in lysis buffer at 37°C for 30 min. The reaction mixture was added to a black 96-well plate (Corning, USA) and incubated at room temperature for 5 min before adding the bacterial lysate. Luminescence was immediately measured using a BioTek Synergy H1 microplate reader (luminance mode), with ATP levels normalized to protein content using the BCA assay.

### Infection model and preliminary *in vivo* treatment verification

The neutropenic mouse thigh infection model was used for *in vivo* studies. Specific pathogen-free female ICR mice, 5–6 weeks old and weighing 18–22 g (Shanghai Jiesijie Laboratory Animal Co., LTD), were used in this experiment. Mice were maintained in accordance with the National Standards for Laboratory Animals of China (GB 14925-2010). Neutropenia (neutrophils 100/mm^3^) was induced by injecting cyclophosphamide (Yuanye Biotechnology Co., Ltd., Shanghai, China) intraperitoneally at 4 days (150 mg/kg) and 1 day (100 mg/kg) prior to thigh infection. The mice were infected by an intramuscular injection of 100 µL of exponentially growing bacterial suspension (10^6^–10^7^ CFU/mL) into each posterior thigh muscle.

*In vivo* treatment studies were initiated 2 h after bacterial inoculation. To ascertain the effectiveness of colistin and macelignan combinations, colistin was administered intraperitoneally at 5 mg/kg every 12 h, as monotherapy or in combination with macelignan (100 mg/kg every 12 h). The mice were euthanized after 24 or 48 h of therapy. Bacterial burden was quantified by CFU determination from posterior thigh homogenates. Groups of two mice (four thigh infections) were included in each dosing regimen. Untreated control mice were similarly sacrificed before treatment and at 0, 24, and 48 h after treatment for comparative and evaluation reasons.

### Transcriptome profiling analysis

To study the transcriptomic response of *E. coli* to macelignan, logarithmic phase *E. coli* ATCC 25922 cultures were prepared and treated with macelignan (8 µg/mL), colistin (0.008 µg/mL), or their combination (macelignan 8 µg/mL and colistin 0.008 µg/mL), and incubated at 37°C for 4 h, using a drug-free group as the control. It was placed at 4°C and centrifuged at 4,000 rpm for 5 min to collect the precipitate, then washed twice with PBS, and finally frozen with liquid nitrogen for 30 min before storing at −80°C. Sequencing was performed at Sansu Biotechnology Co., Ltd. (Jiangsu, China) using the Illumina NovaSeq 6000 platform (2 × 150 bp paired-end sequencing).

### RT-qPCR validation of gene expression

Differentially expressed genes identified in the transcriptome were validated using quantitative real-time PCR (RT-qPCR). Logarithmic-phase *E. coli* ATCC 25922 was subjected to treatment with macelignan (8 μg/mL), colistin (0.008 μg/mL), or their combination (macelignan 8 μg/mL plus colistin 0.008 μg/mL), followed by incubation at 37°C for 4 h. Total RNA was extracted from *E. coli* cultures using Magnetic Particles Extract Bacterial RNA Kit (Solarbio, Beijing, China), and RNA concentration and purity were determined using a NanoDrop 8000 spectrophotometer (Thermo Scientific, Waltham, MA, USA). DNase I-treated RNA samples (≤1 µg) were reverse transcribed into cDNA using the HiScript IV All-in-One Ultra RT SuperMix for qPCR (Vazyme, Nanjing, China) according to the manufacturer’s instructions.

Based on the results of the transcriptome analysis, eight target genes were selected (Table 1), and their sequences were obtained from GenBank. Specific primers were designed using Primer 3 Plus (version 0.4.0), and their specificity was verified through BLAST analysis. RT-qPCR was performed on the LightCycler 96 real-time PCR system (Roche, Switzerland) using Hieff qPCR SYBR Green Master Mix (Low Rox Plus) (Yeasen, China). The PCR amplification program included initial denaturation at 95°C for 5 min, followed by 40 cycles of denaturation (95°C, 10 s) and annealing/extension (60°C, 30 s). Relative gene expression levels were calculated using the 2^−ΔΔCt^ method, with *RecA* as the internal reference gene. All reactions were performed in triplicate.

**TABLE 1:** Primer sequences used in this study^*a*^

Gene	Primer sequence (5′−3′)	Source
*btsT*	F: CCTGCATGAAATGGGTGG R: GGATCTGCTCTGGCGAAAT	This study
*torC*	F: CAATTTATGCGGCGAAAGGC R: ATTGGGTGAGTACACGCTGA	This study
*rclB*	F: TCAGCATCACCGACAGTTCT R: ACAATACTGCTGTTGCGGTT	This study
*yjfM*	F: CGCAGCACTTCTGGTGATTA R: GCTGGAAGAACGACCATAGC	This study
*citC*	F: CGTGATGGTGCTGATGGA R: GTAAAGGGATTGGCGTTC	This study
*citX*	F: ACCTGCTTCCTGAACTCGC R: TGACCTCGCTGTCTTTAATCG	This study
*citF*	F: TCAACGACAAGAACGGGTAG R: TGTAAACCAGAGCGACGAAT	This study
*citG*	F: TTTGGTGCCTGTAGTGCG R: GCTGCCTTTATGCGTGTTT	This study
*RecA*	F: GCGCGAAGGTAAAACCTGTG R: AACGTCTACTGCGCCAGAAC	This study

^*a*^ All primers were designed based on *E. coli* ATCC 25922 genome sequence (GenBank: CP009072) and synthesized by Sangon Biotech.

### Statistical analysis

The Student *t-*test and one-way ANOVA, followed by Dunn’s multiple comparison test, were performed to determine statistical significance, using GraphPad Prism 9.0 (GraphPad Software, San Diego, CA, USA). Data are shown as means ± SD. *P* < 0.05 was considered statistically significant.

## RESULTS

### Susceptibility testing

**Fig 1:**
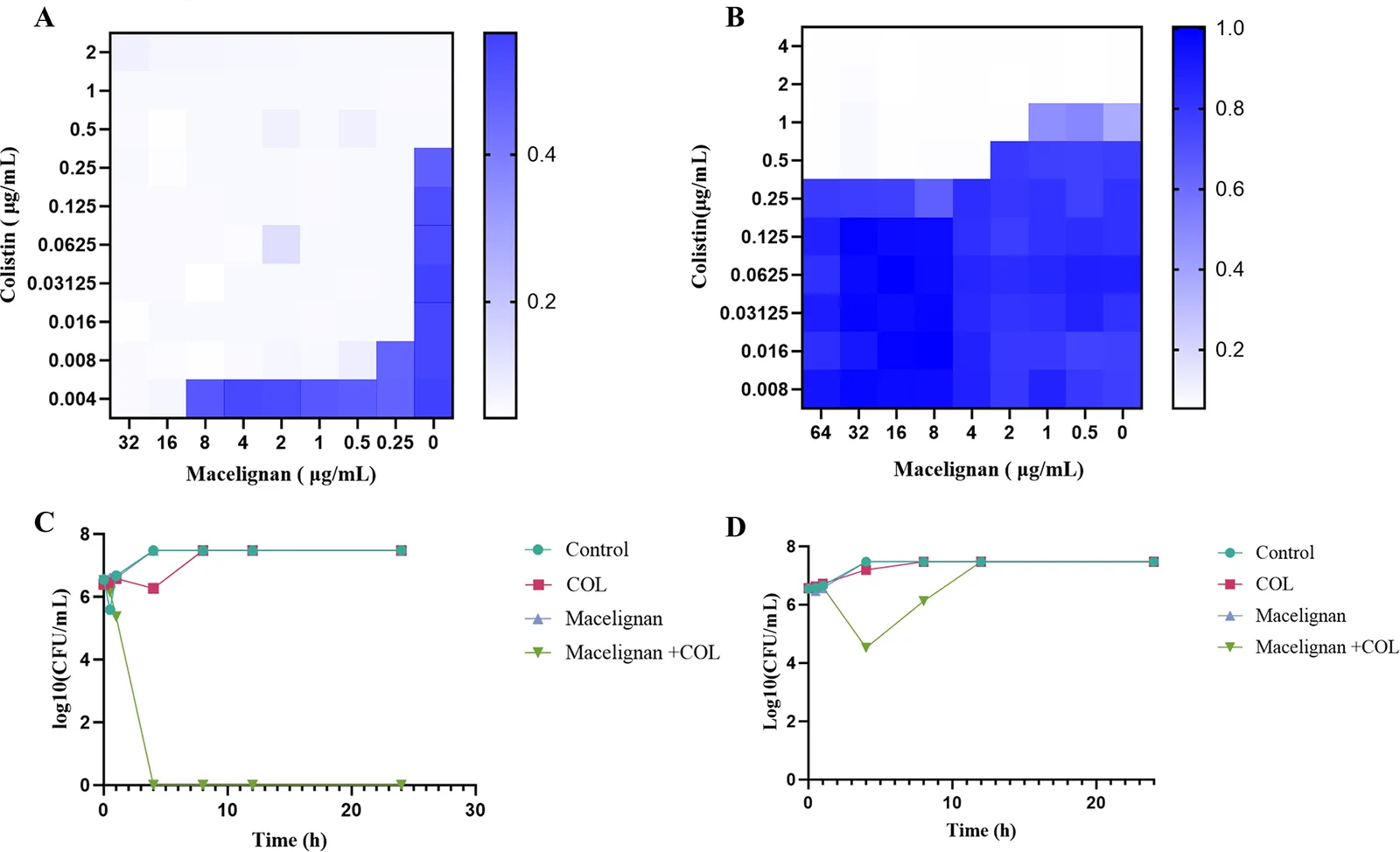
Synergistic effects between colistin and macelignan. (**A**) and (**B**) Checkerboard broth microdilution assays showing the interaction between macelignan and colistin against *E. coli* ATCC 25922 and *E. coli* M17GZZ15, respectively. Time-kill curves for *E. coli* ATCC 25922 (**C**) and *E. coli* M17GZZ15 (**D**) were determined using colistin alone, macelignan alone, or their combination. The concentrations used were 0.008 µg/mL colistin and 8 µg/mL macelignan for ATCC 25922, and 0.5 µg/mL colistin and 64 µg/mL macelignan for M17GZZ15.

The MICs of colistin against *E. coli* ATCC 25922 and drug-resistant *E. coli* M17GZZ15 were 0.25 and 2 µg/mL, respectively. The MICs of macelignan against both types of bacteria were greater than 512 µg/mL, indicating no antibacterial activity. The synergistic effect was evaluated by the checkerboard method. Macelignan and colistin had a good synergistic effect, with FICI values less than 0.5 (Fig. 1A). For the *E. coli* ATCC 25922, the MIC of colistin dropped from 0.25 µg/mL to a minimum of 0.004 µg/mL, which was 64 times lower than that of colistin used alone. For the drug-resistant bacterium M17GZZ15, the MIC of colistin dropped from a minimum of 2 to 0.5 µg/mL, which was four times lower than that of colistin used alone (Fig. 1B).

### Time-killing assays

The *in vitro* synergistic interaction between macelignan and colistin was further assessed by time-killing curves using *E. coli* ATCC 25922 and *E. coli* M17GZZ15. As shown in Fig. 1C, against *E. coli* ATCC 25922, macelignan (8 µg/mL) alone did not affect bacterial growth, while colistin alone treatment (0.008 µg/mL) caused only a transient suppression before regrowth occurred at 8 h. Strikingly, the combination of macelignan (8 µg/mL) and colistin (0.008 µg/mL) exhibited a rapid bactericidal effect, eliminating all bacteria within 4 h and preventing any regrowth throughout the 24 h period. For the *E. coli* M17GZZ15 (Fig. 1D), neither macelignan (64 µg/mL) nor colistin (0.5 µg/mL) alone inhibited bacterial growth. However, their combination resulted in a pronounced antibacterial effect, reducing the bacterial concentration by up to 3 log10 (CFU/mL) compared to the control. These findings reveal that the combination of macelignan and colistin produces a synergistic antibacterial effect against both susceptible and resistant *E. coli* strains.

### Safety evaluation of macelignan and the efficacy of the combination *in vivo*

**Fig 2:**
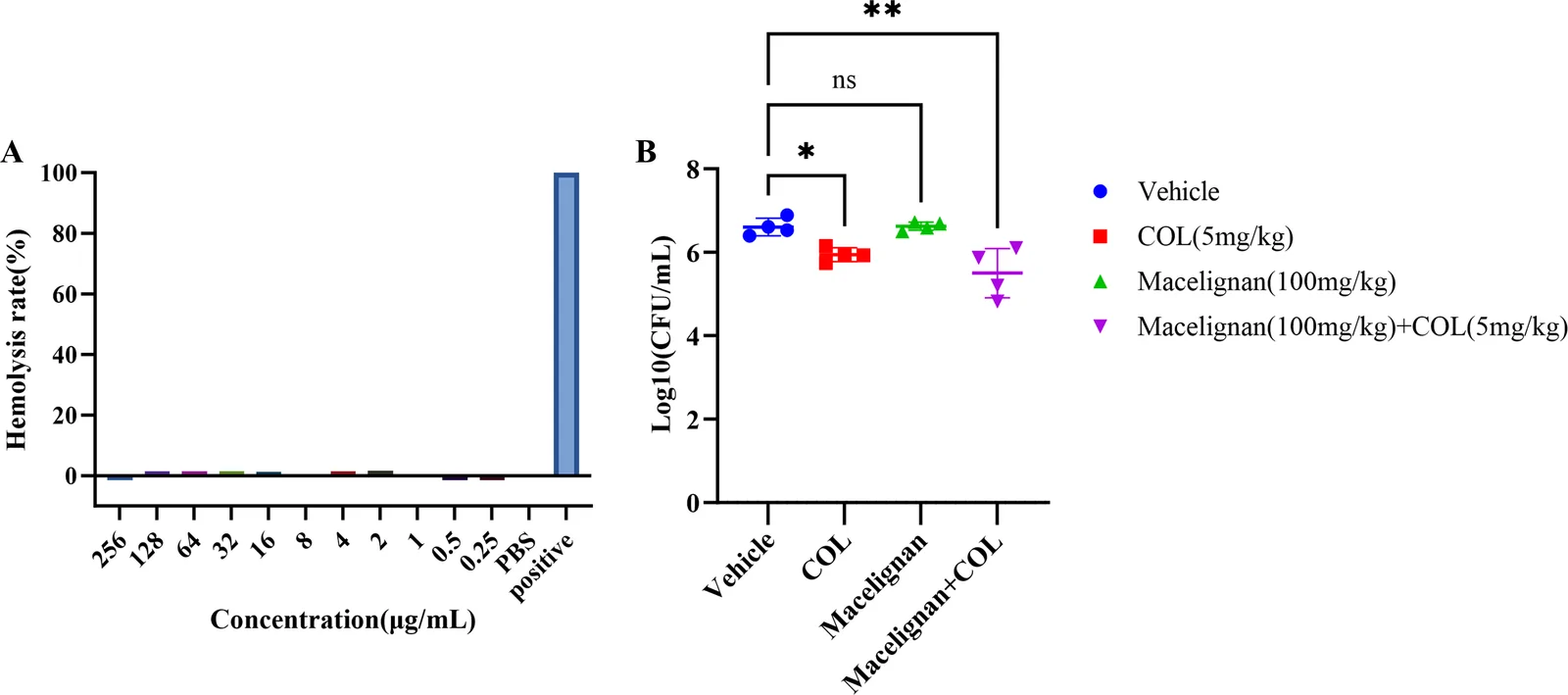
Hemolytic activity of macelignan against rabbit red blood cells (**A**). *In vivo* efficacy of macelignan and colistin, alone and in combination, against *E. coli* M17GZZ15 in a murine thigh infection model after 24 h of treatment (**B**). *P < 0.05; **P < 0.01; ns indicates not significant (*P* ≥ 0.05).

Assessment of the hemolytic potential of macelignan was performed as a critical safety evaluation. As shown in Fig. 2A, the hemolytic rates remained below 0.25% across all tested concentrations, indicating no observable hemolytic activity.

To evaluate the *in vivo* efficacy of the macelignan-colistin combination, we employed a murine thigh muscle infection model. As shown in Fig. 2B, monotherapy with macelignan failed to control infections caused by *E. coli* M17GZZ15. In contrast, colistin monotherapy significantly reduced the bacterial load in thigh muscles compared to the positive control (*P* = 0.0358). Strikingly, the combination of macelignan (100 mg/kg) and colistin (5 mg/kg) resulted in a substantially greater reduction in bacterial burden, demonstrating a highly significant difference from the positive control (*P* = 0.0012). These findings indicate that macelignan can markedly enhance the *in vivo* antibacterial activity of colistin while reducing its required dosage, presenting a promising strategy to combat resistant Gram-negative bacterial infections.

### Effect of macelignan and colistin on the integrity and permeability of bacterial cell membranes

**Fig 3:**
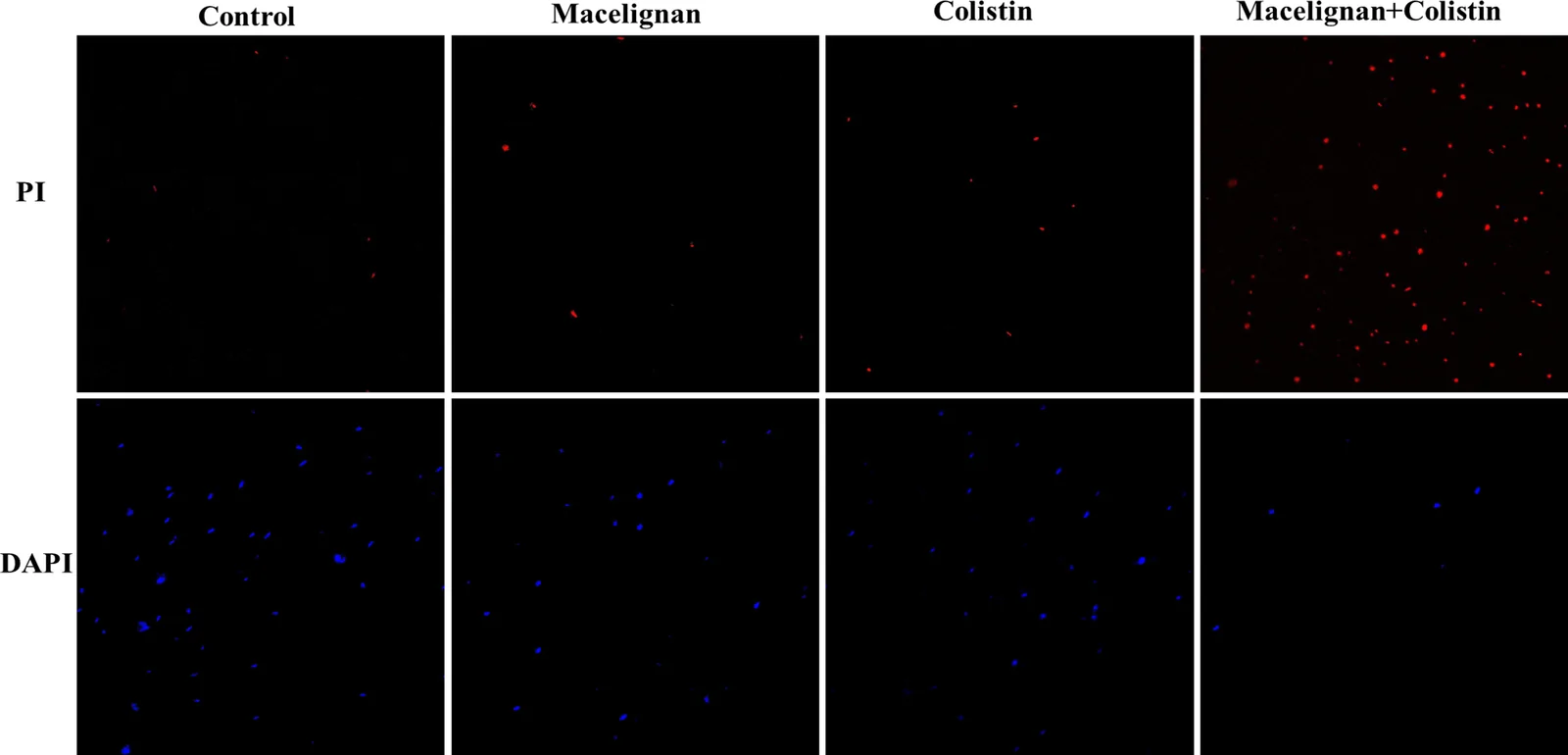
Fluorescence microscopy images of *E. coli* ATCC 25922 stained with PI and DAPI after treatment with colistin (0.008 µg/mL), macelignan (8 µg/mL), or their combination.

The effect of macelignan and colistin, alone and in combination, on bacterial membrane permeability was assessed using the fluorescent probe PI. Treatment with either macelignan or colistin alone resulted in no significant difference in bacterial mortality compared to the positive control. In contrast, their combination led to extensive bacterial death, as indicated by a substantial increase in red fluorescence (Fig. 3). These results demonstrate that while each drug alone has little effect on membrane integrity, their combination causes significant membrane damage.

The effect of drug treatment on bacterial membrane permeability was further evaluated using DAPI, a membrane-permeable nucleic acid stain that produces blue fluorescence upon binding to DNA in live cells. As illustrated in Fig. 3, robust blue fluorescence was detected in the positive control and single-drug treatment groups, suggesting that the majority of bacteria remained viable with intact membranes. However, combined treatment with macelignan and colistin led to extensive bacterial death, accompanied by a dramatic reduction in blue fluorescence. These results indicate that the synergistic bactericidal activity of macelignan and colistin is mediated through disruption of bacterial membrane integrity.

### ROS and ATP levels

**Fig 4:**
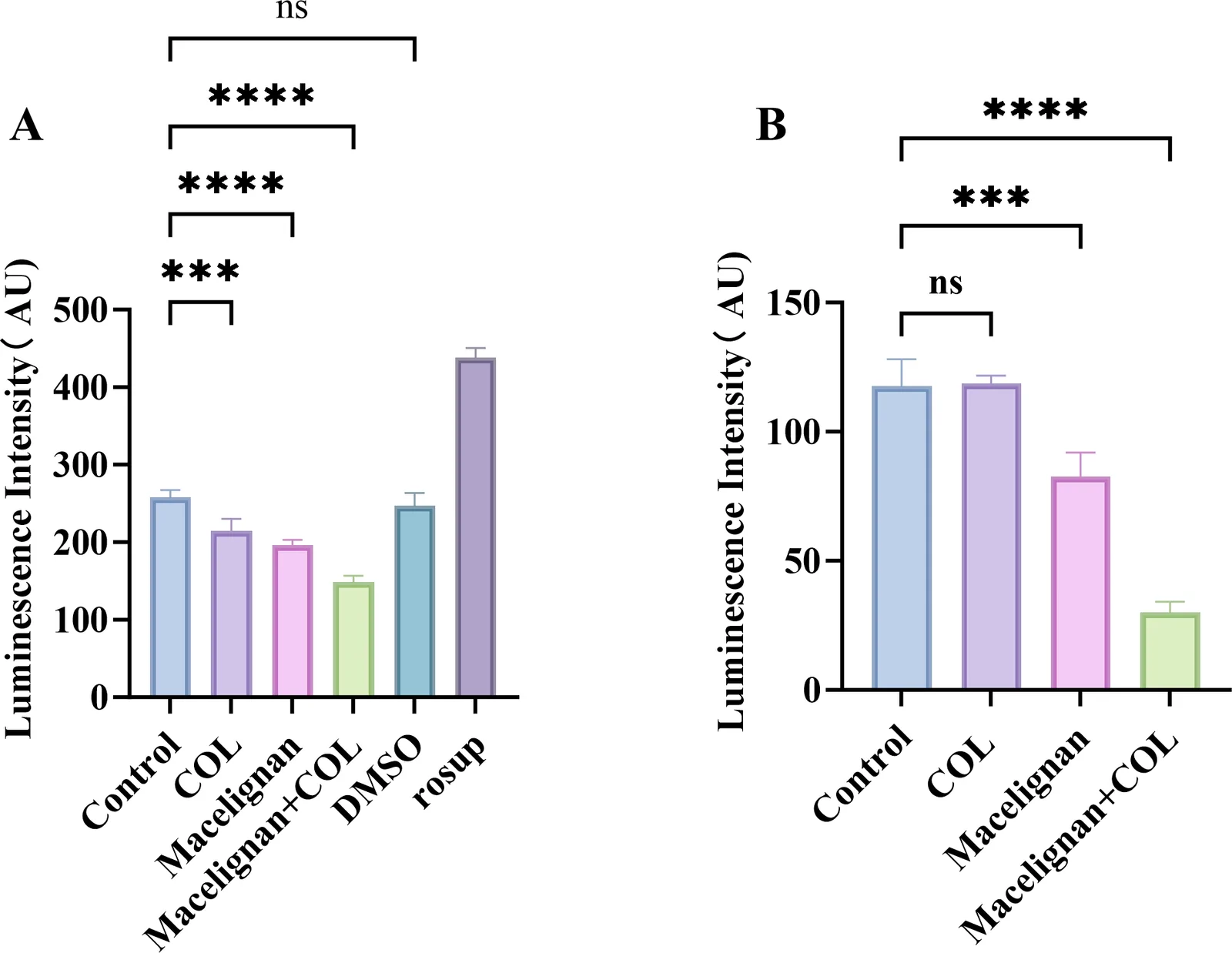
Effects of colistin alone or combined with macelignan (8 µg/mL) on intracellular ROS levels (**A**) and ATP accumulation (**B**) in *E. coli* ATCC 25922. All data are presented as mean ± SD. Statistical significance was determined by one-way ANOVA. ***P < 0.001, ****P < 0.0001, and ns indicates no significant difference (*P* ≥ 0.05).

To comprehensively assess the cellular state following drug treatment, intracellular ROS and ATP levels were measured in *E. coli* ATCC 25922. While treatment with macelignan or colistin alone had minimal effect, their combination significantly reduced both ROS and ATP levels (Fig. 4A and B; *P* < 0.05). This decline in ROS may reflect either the antioxidant activity of macelignan or the rapid loss of bacterial viability. Meanwhile, the marked ATP depletion indicates compromised membrane integrity, leading to metabolite leakage and disruption of energy-dependent processes. These findings suggest that the combination exerts synergistic bactericidal activity by inducing oxidative imbalance and energy depletion.

### Transcriptome analysis

**Fig 5:**
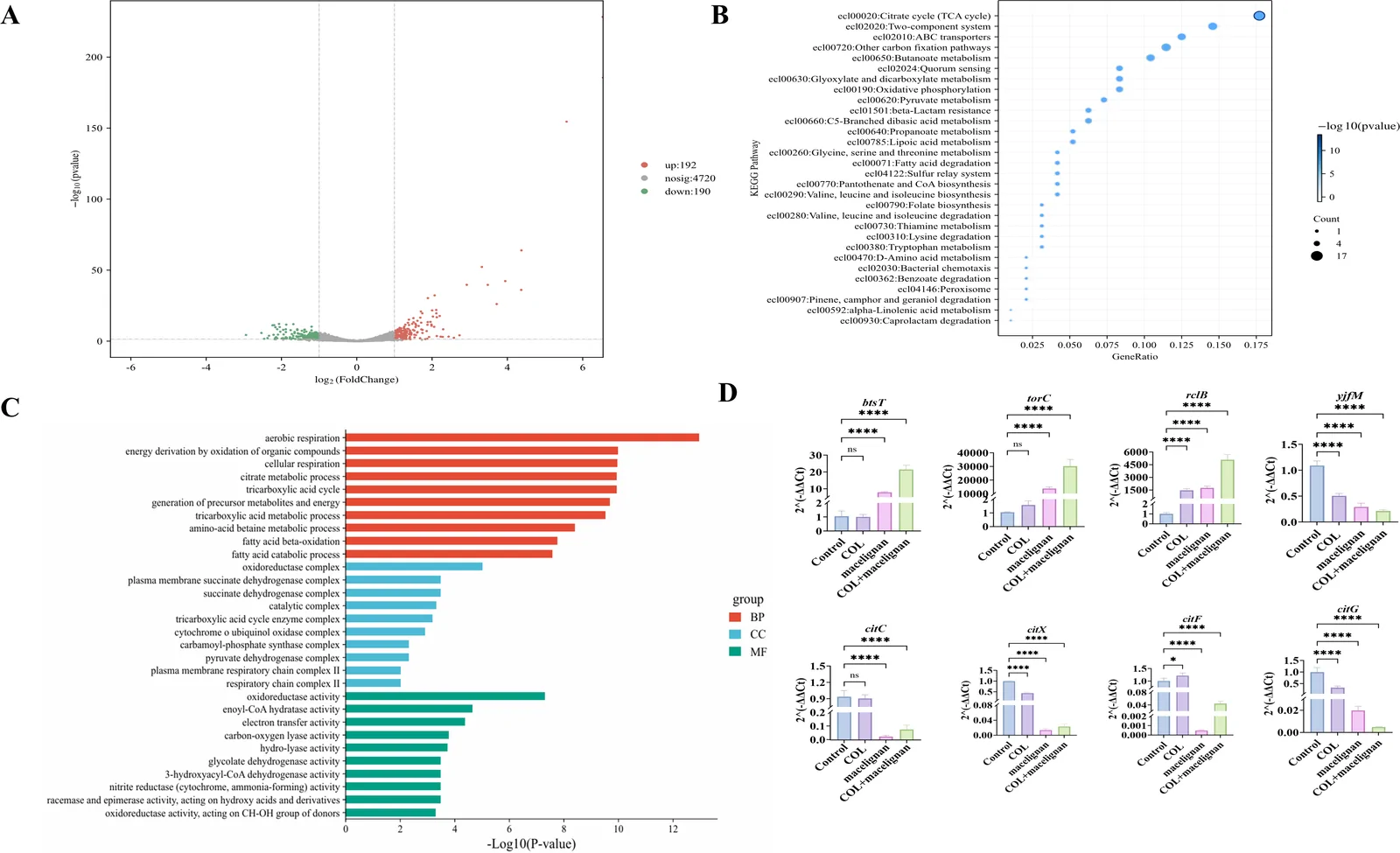
Identification and enrichment analysis of DEGs. (**A**) Volcano plot illustrating the distribution of DEGs in *E. coli* following treatment with the combination of colistin and macelignan. (**B**) KEGG pathway enrichment analysis of the identified DEGs. (**C**) GO term enrichment analysis of DEGs. (**D**) Validation of selected DEGs by qRT-PCR. Data are presented as mean ± SD (*n* = 5). Statistical significance compared to the untreated group was determined by Student’s *t*-test. *P < 0.05 and ****P < 0.0001.

To elucidate the molecular mechanism underlying the antibacterial activity of macelignan, transcriptomic analysis of *E. coli* treated with macelignan was performed. Compared to the untreated control, a total of 382 differentially expressed genes (DEGs) were identified, comprising 192 upregulated and 190 downregulated genes (Fig. 5A). Gene Ontology (GO) functional enrichment analysis of these DEGs revealed that they were predominantly associated with biological processes related to energy metabolism, cellular respiration, and the citric acid cycle (Fig. 5C). These findings suggest that macelignan may exert its antibacterial effect by modulating these key metabolic pathways.

KEGG pathway analysis revealed significant enrichment of DEGs in the citrate metabolism pathway (Fig. 4B). Specifically, genes involved in citrate utilization (citC, *citX*, *citF*, *citG*) were markedly downregulated upon macelignan treatment, suggesting a blockade of the citric acid cycle. This transcriptional suppression aligns with the observed reduction in intracellular ATP levels (Fig. 4B). Concurrently, the upregulation of stress-related genes (torC, *rclB*) and metabolic transporters (btsT) likely reflects a bacterial stress response to energy depletion and metabolic imbalance. These findings collectively support a model where macelignan disrupts central carbon metabolism, depleting cellular energy stores and thereby compromising the bacterial capacity to repair colistin-induced membrane damage.

## DISCUSSION

The widespread use of antibiotics in livestock and poultry breeding has accelerated the rapid development of bacterial resistance, leading to a rising incidence of MDR and extensively drug-resistant infections in humans (21–23). Among these, MDR Gram-negative bacteria pose a particularly serious threat (24). Despite the urgent need for novel antibiotics to combat these infections, no new classes of Gram-negative antibiotics have been introduced since the discovery of quinolones (25). Consequently, the development of new antibacterial agents or the identification of safe antibiotic adjuvants has become a global priority. Combination therapy has emerged as a promising strategy to overcome antibiotic resistance (9, 26). Colistin, first discovered in the 1940s (27), was largely abandoned in the 1970s due to dose-dependent nephrotoxicity (28, 29). However, the recent resurgence of MDR Gram-negative infections has renewed interest in colistin, with a particular focus on identifying adjuvants that can enhance its antibacterial efficacy and reduce the required clinical dose, thereby mitigating its associated toxicity.

A key finding of this study is that macelignan synergistically enhances colistin activity, allowing for dose reduction and potentially minimizing colistin-associated toxicity. Macelignan, a bioactive lignan derived from *Myristica fragrans*, exhibits a broad spectrum of pharmacological activities, including antioxidant, antibacterial, anti-inflammatory, and hepatoprotective effects (15), with recent studies also reporting anti-lung cancer properties (17). Its hepatoprotective mechanisms involve modulation of drug-metabolizing enzymes, such as inhibition of aminopyrine N-demethylase (30), and regulation of MAPK signaling against cisplatin-induced hepatotoxicity (31). Furthermore, *in vitro* toxicity studies have confirmed that macelignan lacks cytotoxic effects on lymphocytes (32, 33), underscoring its favorable safety profile as a colistin adjuvant.

Recently, an increasing number of studies have explored colistin combination therapy with non-antibacterial agents as a synergistic strategy against resistant bacteria (34, 35). Unlike conventional synthetic drugs, compounds derived from traditional Chinese medicine often possess unique physicochemical properties and broader pharmacological profiles (7), making them attractive candidates for combination approaches. Within this context, macelignan emerges as a promising partner for colistin. Furthermore, given that membrane permeability is a critical determinant of antibiotic efficacy, the potential of this combination to disrupt bacterial membrane integrity warrants further investigation.

Antibiotics targeting the bacterial cell membrane are known to exert rapid bactericidal activity (14, 36). In the present study, neither macelignan nor colistin alone exhibited significant antibacterial effects against *E. coli*, likely attributable to limited outer membrane permeability restricting intracellular drug accumulation. However, their combination synergistically potentiated membrane disruption. Time-kill assays demonstrated that combined treatment with macelignan and colistin achieved complete bacterial eradication within 4 h, with no regrowth observed over a 24 h period.

Colistin, a cationic polypeptide antibiotic, exerts its antibacterial effect by binding to the lipid A component of LPS, thereby disrupting the outer membrane of Gram-negative bacteria (37). We propose that macelignan compromises outer membrane integrity—potentially by perturbing lipid organization or inhibiting outer membrane protein function—thereby facilitating colistin access to its target. This proposed mechanism is supported by multiple lines of evidence. First, a significant increase in membrane permeability was observed in the PI uptake assay following combination treatment (*P* < 0.05). Second, DAPI staining revealed a marked reduction in intracellular nucleic acid content, consistent with membrane disruption and leakage of cellular contents. Finally, scanning electron microscopy images demonstrated pronounced morphological damage in the combination treatment group, including membrane collapse, depression, and cavity formation on the bacterial surface.

Macelignan has been reported to protect mammalian cells by scavenging radiation-induced intracellular ROS, reflecting its antioxidant and anti-inflammatory properties (32). In the context of colistin combination therapy, however, we observed strain-dependent differences in ROS levels. The decrease in ROS in *E. coli* ATCC 25922 following combination treatment may be attributed to metabolic arrest resulting from rapid bactericidal activity, rather than direct antioxidant effects. This interpretation is supported by transcriptomic analysis, which revealed significant alterations in the expression of genes involved in the TCA cycle and citrate metabolism following combination treatment—findings corroborated by RT-qPCR. These data suggest that macelignan disrupts bacterial energy metabolism.

Based on these findings, we propose a dual “internal and external pincer” mechanism underlying the synergistic activity of macelignan and colistin. Colistin initiates the attack by binding to LPS and disrupting outer membrane integrity, forcing bacteria to expend energy on membrane repair and homeostasis maintenance. Concurrently, macelignan compromises the bacterial energy supply by inhibiting the citric acid cycle, thereby impairing the cell’s ability to cope with colistin-induced membrane damage. This combination of physical membrane disruption and metabolic depletion ultimately leads to bacterial death—a strategy increasingly recognized as an effective approach to enhance antibacterial efficacy (38, 39).

The translational potential of this combination strategy was further validated in an animal infection model. The bacterial load in mice receiving combination therapy was significantly reduced compared to monotherapy groups (*P* < 0.05). Importantly, by enabling a reduction in the effective dose of colistin, this combination approach may help mitigate its dose-limiting nephrotoxicity in clinical applications.

## Data Availability

All data generated or analyzed during this study are included in this article. No additional data have been deposited in a public repository. The authors certify that they will comply with the journal’s data sharing policy.
